# Behavioural challenges of minorities: Social identity and role models

**DOI:** 10.1371/journal.pone.0220010

**Published:** 2019-07-26

**Authors:** Joseph Vecci, Tomáš Želinský

**Affiliations:** 1 Department of Economics, School of Business, Economics and Law, University of Gothenburg, Gothenburg, Sweden; 2 Department of Regional Science and Management, Faculty of Economics, Technical University of Košice, Košice, Slovakia; 3 Institute of Economic Studies, Faculty of Social Sciences, Charles University, Prague, Czechia; Universidade da Coruna, SPAIN

## Abstract

We present a lab-in-the-field experiment and surveys of marginalised Roma children in Slovakia to examine whether reminding Roma of their ethnicity reduces their performance in a cognitive task. Research on social identity and stereotypes has documented that when individuals feel that their social group is negatively stereotyped in a domain, their performance declines, which can reinforce discrimination. In an effort to break the cycle of negative stereotypes, we remind Roma subjects of either Roma or non-Roma role models. We find that the activation of a Roma’s ethnicity reduces cognitive performance. In contrast, Roma exposed to Roma role models outperform those reminded of their ethnicity and of non-Roma role models. We then attempt to understand the channels through which social identity and role models affect performance. We show that priming the identity of a Roma has a direct effect on confidence, decreasing performance.

## Introduction

Social identity commonly refers to an individual’s own perception of self, based on his or her membership in a group such as ethnicity, race, or gender [1]. A person’s social identity provides a set of rules that govern group behaviour, as such social identity can help to explain behavioural differences across groups [2–7]. An important aspect of one’s social identity are its stereotypes; the physical, mental and psychological characteristics attributed to a typical member of a given social group. Once a set of characteristics are used to describe a social group, those characteristics influence the behaviour of people who are associated with the group [8]. Stereotypes predominately highlight the largest differences between groups as such many stereotypes are exaggerated [9]. For instance, a negative stereotype can generate negative perceptions about one’s self, leading people to perform worse than their abilities would suggest. This is commonly known as stereotype threat [10].

Stereotype threat is especially prevalent in populations that suffer from discrimination. The perceived negative characteristics of these groups in a particular domain tends to influence the group members’ self-beliefs [10]. Negative stereotypes have been found to explain race-based performance differences on academic tests [8, 11, 12]. Asian American women were found to perform better on a math test when their ethnic identity is activated, but worse when their gender identity is activated [8]. A similar stereotype threat was also found when comparing a task titled “sport intelligence” in one treatment and “natural athletic ability” in the second treatment on a sample of black and white college students. Black participants performed significantly worse in the “sports intelligence” diagnostic and, in contrast, white participants performed worse in the “natural athletic ability” diagnostic [13]. Gender stereotypes were also investigated and a study [14] assessed the impact of reinforcing stereotypical information suggesting that women are less competent players on women’s digital gaming performance. The authors found that the stereotype threat condition had a negative effect on performance and affective responses, whereas no differences were observed when comparing the stereotype neutral and stereotype boost conditions. Further, in India, when the stereotypes associated with low caste subjects, who have historically been discriminated against, is made salient, the group’s performance declined compared to that of non-discriminated-against high caste subjects [2, 15].

While most of the literature studying stereotype threat focuses on adults, there is a smaller but growing literature using children. This literature shows that children are aware of gender and ethnic categories at a very young age [16], although evidence regarding the identification of their own ethnicity is rather ambiguous [17]. Nevertheless, children as young as 6-7 have been shown to believe that (other) children from high socioeconomic status (SES) are better at school than children from low SES [18]. Similar conclusions were obtained by a recent study [19] investigating the perceptions of stereotype threat by 7-11 years old children. The majority of studies involving a child sample focus on stereotype threat related to gender, in particular, females’ gap in maths abilities. Nonetheless, there is also a stereotype according to which boys are academically inferior to girls, which also was supported by experimental evidence [20]. In a recent meta-analytic study [21] the effects of stereotype threat on performance of girls in maths, science and spatial skills tests were studied. The authors identified signs for the presence of publication bias which may significantly distort the literature on the effects of stereotype threat among school-age girls. This is in accordance with the suspicion of a previously published study [22] that used implicit and explicit methods to activate stereotype threat, but found no impact on school-age girls’ mathematics performance. The study thus suggests that stereotype threat either occurs all the time or only in very specific circumstances.

The evidence for stereotype threat related to children’s ethnicity and race has also been supported by numerous studies in contexts such as: the contribution of diagnostic testing to the achievement gap in African American students [23]; effects of stereotype threat on African American, American Indian, Latinos, Asian American, and European American children’s connection to school [24]; or the negative effects of stereotype threat on the school success of minority adolescents from Turkish and Moroccan backgrounds in Belgium [25].

Negative stereotypes can be particularly harmful as they can reinforce discrimination across groups. A negative stereotype increases negative thoughts about oneself and can reinforce a negative identity. In turn, this can directly affect confidence, aspirations and levels of effort expended by the individuals or groups, ultimately affecting their learning and performance, reinforcing negative attitudes towards the group, and terminally discrimination [26]. A key component of this cycle is that negative stereotypes not only influence how a group member perceives him/her self, but also how others perceive the social group. For example, research has found that negatively stereotyped names can reduce an employer’s effort to inspect resumes [27], and influence wages and employment opportunities [28]. See, e.g. [29–31] for further discussion on statistical discrimination and related literature. Thus, understanding the factors that influence group stereotypes and determining how stereotype threat can be reduced is important in countering discrimination directed towards social groups.

In this paper, we examine the effect of role models on social identity and, in particular, on social group stereotypes of Roma children. This is a particularly important in contexts, where, for instance, social identity and skin colour stereotypes affect the aspirations and performance of students [32], as previous research shows that numerous Roma students do not aspire to secondary education, which is also attributed to the lack of role models [33]. We begin by investigating whether reminding members of a negatively stereotyped ethnic group of their ethnic identity affects their academic performance. Negative stereotypes are often numerous and may be continuously activated throughout an individual’s day. In an effort to break the cycle that negative stereotypes may perpetuate, we examine the activation of ethnic and non-ethnic role models on academic performance. Reminding people of their role models, individuals who are perceived as worthy of emulation [34], may remind subjects of a positive aspect of their social identity, offsetting negative self perceptions, improving performance, and thereby lessening the effects of negative stereotypes [5]. Research on social identity confirms that similarity between self and others increases the likelihood of social comparison [1]. By comparing ethnic role models to non-ethnic role models, we can also identify whether role models who are similar to participants in terms of ethnicity and perhaps upbringing have a different effect on subjects’ behaviour than more distant role models students may not personally relate to. Lastly, we attempt to understand the channel through which negative stereotypes and role models affect performance. We hypothesise that priming a subject’s ethnic identity may have a direct effect on confidence, resulting in a decrease in performance. Therefore, we test whether confidence changes when a subject’s ethnicity is made salient and whether being reminded of role models can offset any reduction in confidence.

Other common interventions aimed at offsetting the negative effects of stereotype threats implemented by researchers include, for instance, mentoring programmes. A field experiment [35] targeting female, minority, and low-income adolescents was shown to have significant positive impacts on math standardized test scores of all three investigated groups (in comparison to control conditions), thus reducing the stereotype threat consequences. Creating an environment of perceived equal treatment of students of different ethnicities has also been shown to buffer negative effects of discrimination and stereotype threat [25].

We employ an artefactual field experiment with 394 Roma children in Eastern Slovakia. Roma are the largest minority in Europe and, according to the EU, they suffer from widespread discrimination [36]. In a recent report, Roma were found to be the minority discriminated against second most often, with 26% of Roma reporting experiencing discrimination, preceded by North Africans (31%), and followed by Sub-Saharan Africans (24%), Turks (20%), South Asians and Asians (10%), recent immigrants (10%) and Russians (6%) [37]. This translates into Roma’s belief that negative attitudes exist towards them in education, housing, and employment [38]. This context provides a unique opportunity to study the effects of social identity and whether reminding Roma of their role models can help to mitigate the effects of negative stereotypes on academic performance.

Similar to [4, 8, 39–41], we use a pre-experiment background questionnaire to make Roma ethnicity salient. Participants then take part in a simple math task to gauge cognitive performance. In two additional treatments using the background questionnaire, participants are reminded of Roma or non-Roma role models. We find that the activation of a Roma’s ethnicity reduces cognitive performance compared to the control of no ethnic or role model information. In contrast, students reminded of Roma role models outperform students reminded of their ethnicity and non-Roma role models, but they perform similar to the control group. We thus find that the reduction in performance when children are reminded of their identity can at least partially be attributed to a reduction in confidence. Our findings suggest that reminders of Roma role models can potentially mitigate this decrease in confidence.

The effect of role models on student outcomes has been investigated in a number of empirical studies. This research suggests that role models can significantly influence outcomes, as they have the potential to motivate individuals and serve as a source of inspiration [42]. In this regard, numerous studies estimate the racial interactions between students and teachers [43–46]. They find a positive effect of similar race teachers on various student outcomes. Another study [47] uses survey data to examine whether minority students benefit from taking courses with a same-minority lecturer. The authors show that the performance gap in terms of class drop-out rates and grade performance between white and minority students was smaller when the minority students were taught by minority instructors. Bettinger & Long [48] investigate the impacts of policies designed to increase female representation on college faculties. Their research shows that female instructors positively influence course selection and major choice in some disciplines. Recently, Lusher, Campbell & Carrell [49] use administrative data from a large, diverse university in California, their results show a positive and significant increase in course grades when students are assigned teaching assistants of a similar race/ethnicity. However, ethnic “matching” in their setting may be too simplistic, as it does not necessarily take into account minority students’ perceptions of such teachers, and students may not recognise them as role models [50]. Another study [51] used a representative survey data of middle school students in China and they show that female teachers alter girls’ beliefs about commonly held gender stereotypes, increase their motivation to learn, and ultimately, raise girls’ test scores and improves their mental status and social acclimation relative to those of boys. In contrast to commonly reported results, Puhani [52] uses a German administrative panel and shows only a very weak effect of teacher gender on either recommended or chosen school type for either boys or girls. He also finds no effect of elementary school teacher gender on either subsequent changes in school type or grade repetition. In part, such results can be explained by omitted variables bias, as pointed by [53]. While [10] examines whether Barack Obama had a positive effect on the achievements of African Americans, he finds little difference in a range of outcomes including crime rates and labour force participation between African Americans and whites. Further, role models have been shown to have an effect on adolescent consumer purchase intentions and purchase behaviour [54], as well as on adolescents’ attitudes toward violence and violent behaviour [42]. Related is also the stream of literature on leaders, who have the potential to act as role models and they can influence people’s behaviour in social domains like charitable giving, tax evasion, corporate culture, and corruption [55].

There is a large psychology and growing economic literature on social identity, and, separately, on role models. Our work differs from this research in four important ways. First, while there is a large body of work identifying the importance of social identity and similarly a large body of literature on understanding the impact of role models, the literature bridging these two fields is relatively rare. In this respect, the papers most similar to ours are the novel works [56, 57]. Study [56] investigated the effect of role models when role models are perceived to be doubtful (i.e., express doubt about their abilities). The authors show that doubtful in-group role models hurt females, but were beneficial for males, while out-group role models had no differential effect on performance. Further, increasing the similarity of highly competent in-group role models reduces the stereotype threat experienced by females [57]. These studies investigate the effects of in-group and out-group role models in a gender-stereotyped setting, while this paper assesses the effects of in-group/out-group role models on buffering the academic performance of negatively stereotyped Roma, i.e. stereotype threat based on ethnicity rather than gender. The two settings differ considerably. Clearly, in most societies women and men interact regularly, whereas, segregated ethnic groups such as the Roma (among others) often limit their interaction with the majority population [58]. Further, unlike the current literature, we identify the participants’ actual role models, and then prime those role models, rather than the current procedure in the literature which involves researchers selecting people who may act as role models, such as teachers, and then testing if a role model effect exists. Unlike the current literature we also incentivise the experiment.

Second, our experimental approach allows us to explicitly observe and identify the effects of role models separately from other social and environmental factors. This is particularly difficult in empirical studies because many factors that influence student and role model interactions tend to be unobserved. For example, without an experiment it is difficult to isolate whether effects are due to students or teachers behaving differently. Third, we add to the literature by investigating confidence as a possible channel through which negative stereotypes and importantly role models affect behaviour. By influencing belief in one’s social group, role models may directly affect confidence. Credible empirical evidence on the effect of role models on self-confidence is rare, due to the difficulties of measuring and collecting data on confidence. Improved self-confidence has been found to impact: motivation [59]; firm outcomes [60]; labour market outcomes [61]; wage rates [62]; and the persistence in intergenerational income and educational inequality [63]. Finally, in this paper we study the behaviour of an understudied but important group—the Roma community in Europe. Roma are the largest minority in Europe, and experience far lower living standards than other groups. Approximately, 20% never finish a single grade of primary school, less than 30% of European Roma are in paid work, and 87% live below the national poverty line, according to the European Union Agency for Fundamental Human Rights [64]. To overcome deprivation and bolster inclusion, the EU has spent over 7 billion euro since 2000 on inclusion and anti-poverty programs aimed at Roma [65]. We investigate the effectiveness of an inexpensive program that has the potential to bolster inclusion and increase academic performance.

## Experiment design

Ethics approval was obtained from the Dean of the Faculty of Economics, Technical University of Košice, as the University does not have its own Institutional Review Board. Participation in the research was conditional upon parents’ signed consent on behalf of their children. Data was collected and analysed anonymously.

Upon commencement of the experiment, subjects responded to a “background questionnaire” that varied by experimental treatment [see S1 Appendix]. The experiment consisted of a control in which neither ethnicity nor role models were made salient, and three treatments: 1. Roma ethnicity salient (Roma salient); 2. Roma role model; 3. non-Roma role model. Following [4, 8, 39–41], we use a background questionnaire to make identity and role models salient, because this creates saliency without explicitly reminding the subjects of their ethnicity, thus reducing the probability of an experimenter demand effect.

In the control, neither stereotype or role models were made salient. The background questionnaire consisted of 5 nondescript background questions, such as the subject’s favourite food, favourite colour etc. The questions were unrelated to identity or role models. In the Roma ethnicity salient treatment, the background questionnaire included the same five simple questions asking subjects about their favourite food and colour, followed by three questions related to their ethnicity. These were: their self-declared ethnicity, language spoken most frequently at home and whether their grandparents spoke any language other than Roma. It is important to note that Roma children are often exposed to the non-Roma majority throughout societal interactions, in particular at school, where the majority of teachers are non-Roma. Thus, it is entirely plausible that their identity may be salient in many daily activities, making the Roma salient treatment a plausible representation of reality. Further, in the background questionnaire we use the term “nationality” instead of the term “ethnicity”, which would be difficult for children to understand. With the reference to The Cambridge Dictionary [66], one of the meanings of nationality is “a group of people of the same race, religion, traditions”. In addition, the Slovak language strictly distinguishes between nationality in the context of ethnicity [*národnosť*, which is the literal translation of the term nationality] and nationality in the context of citizenship [*občianstvo*].

The background questionnaire in the Roma role model and non-Roma role model treatments did not directly include questions on ethnicity, but consisted of six simple questions regarding famous Roma/non-Roma role models. In this study, we define role models as nonparental adults who adolescents look up to and want to emulate. To gather information on the people Roma children look up to, we surveyed and informally interviewed 50 Roma adults and children, as well as teachers and community social workers two weeks prior to the experiment. Each interviewee was asked to list the people they most look up to, or that they think Roma look up to. A list was then compiled of the most popular Roma and non-Roma. In order to cover a broad group of role models, we selected popular male and female role models from a range of occupations. The following people were selected as Roma role models: Vladimír Oláh, a poet who established a Roma cultural association in Slovakia; Dr Ján Cibuľa, an activist who was nominated for a Nobel Peace Prize and who was a past president of the International Roma Union; and four popular Roma Slovak or Czech singers/performers. These were: Silvia Šarköziová, the lead female vocalist in the Gypsy Devils band; Igor Kmeťo, the front man of the popular band Kmeťoband; female vocalist Věra Bílá; and the members of a male Slovak band, Gipsy Kajkos. The participants were asked questions such as the maiden name of Věra Bílá, the number of band members in Gipsy Kajkos, the name of Silvia Šarköziová’s band, and the association established by Vladimír Oláh. The survey did not explicitly reference the role models’ ethnicity, however, with reference to shared group membership [67, 68], we believe that this treatment also primed ethnicity. As such comparison between the Roma role model treatment and Roma salient treatment will reflect the effect of Roma role models. We selected this design as we believe it is representative of reality. It would be very difficult to separate the two effects—Roma identity salience and Roma role models as one cannot make a Roma role model salient without also making Roma identity salient. In both treatments (Roma salient; Roma role models), our aim was thus not to remind the subjects of their ethnicity directly.

To ensure that the role models were comparable across treatments, we selected the four most popular non-Roma musicians and two non-Roma role models from other fields. Similarly, we ensured there were two female non-Roma role models. The non-Roma role models consisted of two football players, Cristiano Ronaldo and Marek Hamšík, from Portugal and Slovakia respectively, and four singers/performers; Justin Bieber (Canada), Shakira (Columbia), Helena Vondráčková (Czechia) and Karel Gott (Czechia)—the latter two having been popular in Slovakia (and formerly Czechoslovakia) for decades. Questions included the soccer clubs of the players, the age of Justin Bieber and whether the singer Karel Gott has been awarded “The Golden Nightingale” prize more than 20 times.

To ensure the icons selected are actually role models of the subjects, as part of the post experiment survey we asked whether subjects admire the people mentioned in the background questionnaire. In the Roma role model treatment, 88% of subjects and 94% in the non-Roma role model treatment report looking up to the people/groups mentioned. This is consistent with the existing literature according to which entertainers/athletes are amongst the most frequently chosen role models by children [69] and suggests that the experiment does focus on people the Roma children look up to. This paper differs from a large part of the literature selecting people such as teachers as role models and then testing if a role model effect exists [70].

After participating in the background questionnaire, subjects took part in a numeric task. Participants were given 65 strings of numbers, each string containing between 10 and 20 digits [S2 Appendix]. Subjects were given 3 minutes to count the zeros in each string. For each correctly solved puzzle, the children received 5c. We selected this task as it required mathematical ability, yet it was simple enough for children who have not completed primary school to understand. Roma children are often stereotyped as having low academic ability, and their numeracy skills are perceived to be lower than those of the average population (see the next section). Further, the task was labelled as a “numeric maths task” to emphasise the task’s mathematics nature. The instructions included an example practice string of digits that subjects completed before they began the actual task.

After understanding of the instructions had been checked, but prior to the commencement of the numeric task, a measure of confidence was elicited. We examine confidence as a possible channel through which identity and role models affect behaviour. Similar to [71, 72], subjects were asked to predict their own performance in the task. More specifically, we follow [72] and ask about confidence before the numeric task, to avoid actual behaviour influencing a subject’s guess. Subjects were asked to estimate the number of numeric puzzles they expected to complete in 1 minute. Then, in the post experiment survey they were asked to elicit relative confidence: *If you were to compare your performance with everyone else in this community, how would you rate your performance in comparison to other people?* Possible answers were: *“among the best, better than average, same as average, worse than average and among the worst”*. The latter question was asked in the post experiment survey in order to avoid priming ethnicity.

## Setting and municipality selection

Roma children are the subject of our study. We specifically selected early adolescent children, as researchers have found that as children enter adolescence, they increasingly focus their attention on nonparental adults to identify models they want to emulate [73, 74]. We study Roma, as they are the largest minority in Europe and, according to the European Committee of Social Rights [75], they suffer from pervasive historic discrimination, which has further risen during the economic crisis. In EU Member States, 85% of Italians and 66% of French hold unfavourable views of Roma. In Greece, Britain and Poland, about half hold a negative view of Roma, and 40% hold this view in Germany and Spain [76]. In the context of education, around 60% of non-Roma Slovak students reported an objection when asked to share a desk with a Roma student, and almost 50% report bad or very bad experiences with Roma people [77].

Roma minority are often stereotyped (by majority populations) as wild, noisy, dirty, and passively adapting to situations [78]. In particular, the prevailing stereotypes about Roma include low education and lowered intelligence skills, their negative attitudes to work, misuse of the social system and dependence on social benefits, high fertility rate, inability and unwillingness to adapt to the standards of the majority society [79]. According to a recent study based on a social constructivist theoretical framework, Slovak Roma were perceived by the majority population as “irresponsible dependants and deviants not valuing education and incapable of making wise decisions about their lives” [80, p. 71]. The negative stereotypes about Roma related to their cognitive abilities are often based on their educational outcomes.

In Europe, approximately 20% of adult Roma have attained higher than primary education [81], Roma children are five times less likely to attend compulsory primary education compared to the majority populations and 87% of the Slovak Roma population aged 18-24 leave school without completing secondary education and early school leaving rates of Roma range between 72–98% [82]. Furthermore, between 80 and 95% of Roma speaking students sampled in an international PISA survey did not acquire basic cognitive skills and competencies [83]. Besides, in Slovakia, around 91% of Roma people are at risk of poverty, with only 21% in paid work, and similarly, 87% of Roma in the EU are at risk of poverty, and only 35% of men and 21% of women are in paid work [64].

Such stereotypes may be internalized by Slovak adolescents, who are the least tolerant towards Roma—39% of respondents consider Roma to be “inferior people”, while 5.9% of students studied considered Africans, and 6.5% considered Asians to be “inferior people” [84]. Negative attitudes towards Roma are not only expressed by students, but Roma students are also negatively perceived by teachers. According to a study on ethnical attitudes of teachers in Slovakia [85], surveyed teachers held tremendously negative attitudes towards Roma (mean score of 6.3 on the 7-point Bogardus social distance scale, where category “7” represents “Would exclude *[Roma]* from entry into my country”). Similar attitudes were expressed by the general Slovak population, of which two thirds held an aversion towards Roma [86]. The negative stereotypes about Roma have further been fostered by media, which often present Roma as criminals or asocial people [87]. These tendencies are confirmed in our post experiment survey, which asks subjects “How often do you hear your classmates saying bad things about Roma?”, 41% report every day, 13% of respondents report at least once a week, while 22% report at least once a month. This suggests perceived negative perceptions towards Roma children exist within these communities.

The experiment was conducted in November 2015, in 15 municipalities in Eastern Slovakia, with Roma children living in segregated communities (on the outskirts of a municipality). It is estimated that Eastern Slovakia accounts for 85% of the total Roma population living on the outskirts of municipalities in Slovakia.

Using the 2013 Atlas of Roma Communities in Slovakia [88], we randomly selected 15 municipalities in four districts in Eastern Slovakia. The Atlas is a census of the Roma community initiated by the Slovak government and UNDP, aimed at monitoring the living conditions of the Roma population. Information was collected on all communities in Slovakia with 30 or more Roma people. Column 1 in S1 Table presents the average characteristics of the sample communities, while column 2 presents average characteristics for Eastern Slovakia municipalities (with Roma communities on the edge or outskirts). The results show that there is no statistically significant difference between the sample municipalities and other Eastern Slovakia municipalities in terms of characteristics including percentage of households with access to water, public sewer systems, household electricity/gas, and distance to kindergarten/primary school and physician. Distance to the closest general practitioner and pediatrician show a small statistical difference. All selected municipalities are not further than 30 km from the regional capitals, the cities of Košice and Prešov (see S1 Fig). To aid in comparing community characteristics, in S2 Table we report the average characteristics of our sample municipalities (including Roma and non-Roma) taken from the 2011 census and compare them to Eastern Slovakia and Slovakia. As expected, our sample municipalities contains a much higher rate of Roma than municipalities in either Eastern Slovakia or Slovakia in general.

### Participant recruitment and procedure

To recruit participants for the experiment, community representatives were contacted by telephone and/or email, and asked for permission to conduct research in their community centre. In each municipality, one local social community worker was then hired to invite and recruit individual households. The social workers were either employed with an NGO (ETP Slovakia—www.etp.sk) working on development programmes in the Roma communities, or were employed by municipalities. Social community workers were responsible for visiting Roma families and providing counselling services to them on a daily basis. Because of this relationship, a high level of trust towards them exists. Each social worker was trained in how to select and invite participants, were never informed about the topic of the research. The social community workers were directed to randomly select households from different parts of the municipality the day before the actual experiment. Community workers approached households, informing them that they could participate in research the following day with the possibility of earning some money, and that a session would last 60-90 minutes. Within each household unit, we invited both parents (or only the mother/father in single parent households) and two randomly selected children above the age of 8. There were 19 such cases in which more than two children from the same household participated. Upon agreeing to participate, a contact number for each household was recorded. On the morning of the experiment, participants were informed of the time of their session, some of which were advised they would serve as alternates. Only children who were able to read, write and understand basic math were eligible to participate.

Each session included four children from at least 2 households. Between four and six sessions took place in each municipality, cumulating in between 15–47 participants per municipality and 394 subjects in total. Usually four to six sessions took place in each municipality. In cases in which more suitable rooms were available, the number of sessions was higher. In the case of two municipalities, the experiments took place twice (with a three-week delay), though people from different communities were selected both times. We do not believe that information spillovers were a significant issue due to the task being explained to participants as a simple maths quiz, reducing the likelihood of participants identifying the research questions being investigated.

Experiments took place in the municipal community centres. Upon arrival, the participants were screened for eligibility and the consent form read aloud, and a hard copy was then signed by the parents. Adults then moved to a separate room, where they participated in a separate experiment. Upon entering the room, each child was placed at a desk and assigned a unique ID in order to ensure their anonymity. Each participant received their own envelope with answer sheets with their unique participant ID. Data was entered and checked by two research assistants. Three pilot rounds were conducted prior to the experiment (pilot data is not included in the sample).

### Treatment balance

S3 Table lists the demographics of the average subject in our experiment. Column 1 presents the full sample means, columns 2—5 report the averages by treatment where “C” refers to the control, “T1” the Roma salient treatment, “T2” the Roma role model treatment, and “T3” is the non-Roma role model treatment. Participants in the sample are 13 years old on average and are currently attending regular primary school (66.5%). Within participating households, 70% of the children’s parents are married and 63% have at least one parent that completed primary school. Almost 46% of households have a parent who is employed (full/part-time job or activity in small community services). The mean household income of our subjects is 409 euro per month.

## Results

In this section, we provide details of the experimental results. In order to understand the effect of a subject’s ethnicity on performance, we compare the control to the Roma salient treatment. To examine if subjects reminded of role models have better outcomes, we compare the control to the role model treatments. We compare the role model treatments to the ethnicity salient treatment to identify if reminding children of role models with the same (different) ethnicity reduces negative self perception.

Table 1 displays the average number of numeric puzzles solved, by treatment. Column 4 reports the differences in means and the associated level of statistical significance. In the Roma identity revealed treatment, Roma children solve 8.4% fewer numeric puzzles than when identity is not primed (control). This result is consistent with the negative stereotype threat identified in the literature and suggests that Roma children hold a negative stereotype about their ability in this math task.

**Table 1:** Number of numeric puzzles solved by treatment.

	Treatment	No. of Numeric Puzzles Solved	N	Difference		Statistical test	
						t-test	M-W test
		(1)	(2)	(3)	(4)	(5)	(6)
				C − T1	2.148	0.097	0.106
					(1.288)	[1.668]	[5527]
C	Identity not revealed	27.696	92	C − T2	−0.304	0.813	0.919
		(0.994)			(1.287)	[−0.237]	[4455]
T1	Roma identity revealed	25.547	106	C − T3	2.336	0.105	0.095
		(0.819)			(1.432)	[1.631]	[5242]
T2	Roma role models	28.000	96	T1 − T2	−2.453	0.035	0.055
		(0.818)			(1.157)	[−2.119]	[4291]
T3	Non-Roma role model	25.360	100	T1 − T3	0.187	0.887	0.769
		(1.031)			(1.317)	[0.142]	[5426]
				T2 − T3	2.640	0.046	0.065
					(1.316)	[2.006]	[5532]

Notes: This table shows absolute performance of subjects across treatments (1), and the differences among treatments (4); SE estimates in parentheses. T-test and Mann-Whitney test p-values reported in columns (5) and (6), test statistics in square brackets.

Can role models offset this stereotype threat? Children in the Roma role model treatment solve 9.6% more puzzles than in the ethnicity salient treatment. Making Roma role models salient reduces the negative self-perception of Roma children that appears when subjects are reminded of their ethnicity. However, this role model effect only appears when the role model is from the Roma society, and not when a role model is non-Roma. Subjects reminded of non-Roma role models solve 2.6 fewer puzzles than subjects in the Roma role model treatment. We also find statistical difference between priming Roma role models and non-Roma role models.

**Fig 1:**
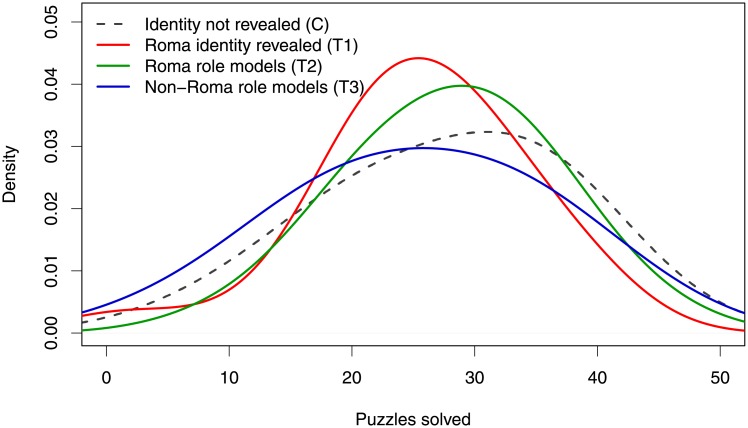
Distribution of numeric puzzles solved by treatments. Note: The figure presents comparison of numeric puzzles solved by control and all treatments (kernel density estimations).

Fig 1 presents the distribution of the number of math puzzles solved, by treatment. Using a Kolmogorov-Smirnov test, the null hypothesis of equality of distributions between the Roma salient and the control is rejected (p–value = 0.012). The mass of the distribution of the math puzzles solved by those in the Roma salient treatment lies to the left of the distribution when identity is not revealed. We also find that the distribution of the Roma role model treatment lies to the right of that of the Roma salient treatment (K-S test, p = 0.060). The distribution of maths puzzles solved by subjects in the Roma role model treatment is similar to those in the control treatment (K-S test, p = 0.275). Finally, we find that the number of math puzzles solved by those in the non-Roma role model treatment lies to the left of those in the Roma role model treatment (K-S test, p = 0.016).

**Fig 2:**
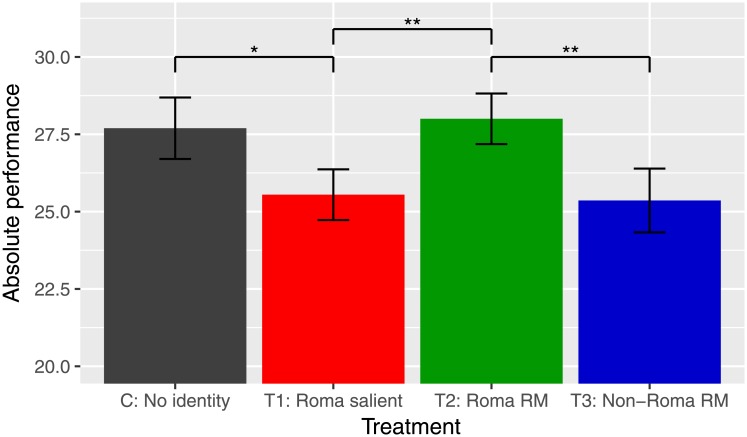
Number of numeric puzzles solved by treatments. Notes: Mean number of numeric puzzles solved by control and treatments. Error bars represent SE estimates. Significance level indications: ***p < 0.01, **p < 0.05, *p < 0.10.

The differences in absolute performance among control and treatments are displayed in Fig 2 suggesting statistically significant differences between the control and Roma salient treatment (t-test, p = 0.097); Roma salient and Roma role models treatment (t-test, p = 0.035); and Roma role models and Non-Roma role models treatments (t-test, p = 0.046).

As a robustness test we examine treatment differences using regressions. Results are reported in Table 2. Column 1 reports results without controls, in the second column we include subjects, household and parental controls, and in the third column we add municipality dummies. The results are consistent with the non-parametric results across all three models. These results use the full sample, despite a small proportion of subjects who stated that they did not consider the Roma and non-Roma role models to be actual role models. Excluding these subjects may bias the result. When we re-estimate our main results in Table 1 excluding those who do not believe the role models are actually role models, we find the number of maths puzzles solved by children in the Roma role model treatment increases slightly to 28.11 while the non-Roma role model group decreases slightly to 25.09. The statistical significance of the results are quantitatively unchanged. Similar results are found when we re-estimate the Table 2.

**Table 2:** Regression of the number of numeric puzzles solved by treatment.

	Dependent: Number of correctly solved puzzles		
	(1)	(2)	(3)
Roma Salient	−2.148*	−2.977**	−2.288*
	(1.288)	(1.276)	(1.280)
Roma Role Model	0.304	−0.190	−0.040
	(1.286)	(1.307)	(1.267)
Non Roma Role Model	−2.336	−2.635*	−2.410*
	(1.432)	(1.365)	(1.329)
Male		−1.137	−0.875
		(0.911)	(0.876)
Age 14 or younger = yes		−2.932**	−2.927*
		(1.457)	(1.514)
Household Income		0.001	0.000
		(0.002)	(0.002)
School = special		−6.418***	−6.255***
		(1.251)	(1.315)
School = other		0.639	1.007***
		(1.752)	(1.783)
Parents employed		2.150**	2.179**
		(0.889)	(0.895)
Parents married		0.662	1.932*
		(1.011)	(1.091)
Father’s edu = unfinished primary		−0.637	−2.281
		(1.340)	(1.604)
Father’s edu = primary		0.338	−0.573
		(1.043)	(1.074)
Constant	27.696***	30.238***	32.660***
	(0.993)	(2.098)	(2.551)
Roma Salient − Roma Role Model	−2.453**	−2.787**	−2.248**
	(1.158)	(1.152)	(1.141)
Roma Salient − Non Roma Role Model	0.187	−0.342	0.122
	(1.317)	(1.231)	(1.213)
Roma Role Model − Non Roma Role Model	2.640**	2.445*	2.371*
	(1.316)	(1.262)	(1.213)
Municipality Dummies	N	N	Y
Observations	394	382	382
R-squared	0.017	0.153	0.244

Notes: This table shows absolute performance of subjects across treatments, and the differences among treatments. Robust SE estimates in parentheses. T-test significance level indications:

*** *p* < 0.01,

** *p* < 0.05,

* *p* < 0.10.

In summary, we find that the negative stereotypes related to Roma identity can have a significant negative effect on achievement, however, this negative stereotype threat can be reduced by reminding children of role models associated with their in-group identity, but not with an out-group identity. However, reminders of role models do not lead to better performance relative to the control.

It is plausible that the number of puzzles solved is related to whether an individual correctly answers the survey questions. This may be problematic if correct answers differ across treatments. To test for this, we compare the number of puzzles solved and responses to the survey questions in the Roma Role model and Non-Roma Role model treatment. If subjects receive a boost from answering survey questions correctly, we would expect subjects in the Roma Role models treatment to answer more questions correctly relative to the Non-Roma Role model treatment. However, we find little robust evidence of this. In fact, in non-Roma role models treatment, subjects score 3.68 survey questions correctly relative to 3.06 in the Roma Role Models treatment (t-test p-value: 0.097; M-W test p-value: 0.106), suggesting that behaviour in the numeric task is not a result of how individuals respond in the survey.

### Mechanisms

The decline in academic performance of Roma children when reminded of their ethnic identity raises an important question—What are the channels through which negative stereotypes affect performance and can the influence of role models impact these channels? One hypothesis is that priming a subject’s identity may have a direct influence on confidence. Role models may improve confidence if participants are primed positively by providing them with an example of someone similar to them who has been successful.

**Fig 3:**
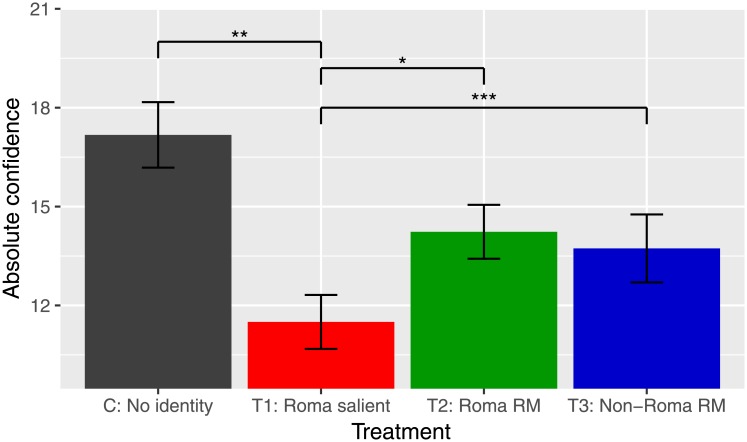
Absolute confidence by treatments. Notes: The figure presents comparison of absolute confidence measure by control and all treatments. Error bars represent SE estimates. Significance level indications: *** *p* < 0.01, ** *p* < 0.05, * *p* < 0.10.

Table 3 (Panel A, columns 1-3 and Panel B, columns 2-3) and Fig 3 present our first measure of confidence—participants’ ex-ante guess of the number of numeric puzzles they expected to solve in one minute. The results demonstrate that subjects’ ex-ante expectation of performance is lowest when they are reminded of their ethnicity. We find that children reminded of their Roma ethnicity expect to perform worse in the task compared to when their ethnicity is not made salient. In turn, subjects in the Roma role model treatment expect to perform better than those in the Roma salient treatment. We also find that the expectation of performance associated with the activation of a Roma role model is similar to that in the non-Roma role model and the control treatments.

**Table 3:** Confidence by treatment.

**Panel A**: Descriptives						
	Treatment	Absolute Confidence			Relative Confidence	Obs.
		Mean	Median	Mode	Percent	
		(1)	(2)	(3)	(4)	(5)
C	Identity not revealed	17.17	10.51	6.16	55.4	92
		(2.03)	(1.34)	(0.90)	(5.2)	
T1	Roma identity revealed	11.50	6.65	4.52	43.4	106
		(1.55)	(0.69)	(0.56)	(4.8)	
T2	Roma role models	14.23	8.91	7.04	64.6	96
		(1.64)	(0.87)	(1.25)	(4.9)	
T3	Non-Roma role models	13.73	10.77	8.47	55.0	100
		(1.14)	(0.77)	(0.87)	(5.0)	
**Panel B**: Statistical tests						
	Difference	Abs. conf.		Rel. conf.		
		M-W test	Median test	Fisher’s test		
	(1)	(2)	(3)	(4)		
	C − T1	0.012	0.025	0.117		
		[5879]	[2.269]			
	C − T2	0.411	0.254	0.234		
		[4625]	[1.209]			
	C − T3	0.813	0.683	> 0.999		
		[4509]	[−0.469]			
	T1 − T2	0.064	0.069	0.003		
		[4230]	[−1.895]			
	T1 − T3	0.001	0.001	0.125		
		[3848]	[−3.339]			
	T2 − T3	0.176	0.086	0.192		
		[4175]	[−1.742]			

Notes: Absolute confidence is based on the assessment of subjects’ estimate of the number of numeric puzzles they expected to complete in 1 minute (*ex ante*). Relative confidence is reported as percentage of subjects who believed that their performance was *“better than average”* or *“among the best”* in their community (*ex post*). Medians and modes reported in Panel A, columns (2) and (3) are based on kernel density estimation. (Bootstrapped) SE in parentheses. Mann-Whitney, Median and Fisher’s exact tests p-values reported in Panel B, columns (2)—(4), respectively, test statistics in square brackets.

Intuitively, we would further expect the absolute confidence level to be positively correlated with subjects’ performance level. The correlation is, however, extremely weak, although positive. We believe a weaker correlation could exist for multiple reasons: 1) If subjects are either very overconfident or underconfident; 2) Since all subjects have never played this numerical task, they have little experience about their predicted performance. We conjecture that since we compare confidence across treatments, these two effects should be mitigated. 3) It is not uncommon in the literature to find low level of correlation between the confidence and outcome [72].

We report our second measure of confidence in column 4 of Panel A and column 4 of Panel B in Table 3. We create a dummy variable equal to 1 if a subject rated their performance as at least better than average compared to other people within their community. Subjects were asked to compare their performance with everyone else in their community. We find that children reminded of Roma role models are more likely to believe they will perform better than others in their community compared to children in the Roma salient treatment. There is little difference across the other treatments. Both measures are at least suggestive that Roma role models have a positive effect on confidence.

## Discussion

The results of this study indicate that reminding Roma children of their ethnicity reduces performance. This result is consistent with the theory of stereotype threat and the previous empirical studies in different cultural settings [2, 8, 10–13, 15, 23–25]. When a negative stereotype is associated with their social group, reminding children of their identity can be detrimental to their academic performance. We find that reminding children of a known role models from their own social group can improve achievement, as opposed to reminding children of role models from different ethnic groups. Our results, based on vicarious role models are hence in line with the results of studies employing role models such as teachers [43–47, 49], as well as the so-called “Obama effect” stream of literature [10, 70].

The desirability of a policy that emphasizes Roma role models is complicated by our finding that reminding children of Roma role models has a similar effect on performance compared to when nothing is highlighted, as in the control treatment. This suggests that not emphasising a child’s ethnicity may also be an important policy option. It is important to note that Roma are often in contact with and exposed to non-Roma (who are the majority); for example, the vast majority of teachers in Slovak schools are non-Roma. According to theories of context dependence [89], the settings in which decisions are made influence behaviour. In this context, the environment Roma are most often exposed to in schools and throughout societal interactions may be more reflective of the Roma salient treatment rather than the control, in which they are not reminded of their ethnicity. The fact that Roma children are in schools exposed to environment where their Roma ethnicity is made salient is also supported by the post-experimental survey data, according to which more than a half of participants reported that their classmates said bad things about Roma every day or at least once a week. Similar unfavourable perceptions of Roma were also reported by surveys conducted among Slovak students [77, 84] or teachers [85]. This would imply the effect of Roma role models on academic performance maybe underestimated when comparisons are made to the control.

We also find that reminding Roma children of non-Roma role models may actually decrease performance in the maths task. It may be possible that role models with little resemblance to oneself may increase the belief that success is unattainable, which may lead to self-deflation decreasing performance. This finding is consistent with literature on shared social categorical self [90] and upward social comparison [91] and similar effects were observed in a study of females exposed to strong/poor-performing female/male peer [92]. When females were exposed to strong-performing female (in-group), stereotype threat effects were eliminated, whereas the exposure of females to strong-performing males (out-group) resulted in an exacerbation of stereotype threat effects. In the light of this study, in our setting non-Roma role models can be thought of as strong-performing out-group models. To understand this mechanism would require further research.

Finally, we investigate confidence as a possible channel through which stereotypes and role models affect behaviour. We find that confidence decreases when subjects are reminded of their ethnicity relative to the Roma role model treatment and the control. This result suggests that the effects of negative stereotypes and role models on achievement at least partially operate through changed confidence. Because confidence plays an important role in behaviour, the effect of negative stereotypes can indirectly impact the outcomes of children in other areas, such as achieving aspirations and employment decisions, which may continue to affect their decisions later in life.

## Concluding remarks

Social identity is an important and growing field of research in economics, in part because identity has a significant influence on behaviour. Its effect on behaviour can be welfare reducing when social groups are negatively stereotyped, such as the Roma group studied here. Roma are among the most socially excluded groups in Europe, facing significant rates of poverty and material deprivation, low levels of education and high levels of long-term unemployment [64, 82]. In order to overcome Roma deprivation and spark Roma inclusion, it is estimated that the EU and local governments spent 3 billion Euro on activities targeting Roma during the 2000-2006 programming period and a further 4.7 billion Euro between 2007-2013 on inclusion and anti-poverty governmental programs and initiatives [65]. Role models may influence social identity and improve confidence and inspire young people to work harder to achieve goals. In the long-term, recognized role models can help to reduce discrimination and provide a cost effective tool to aid inclusion. This paper breaks new ground by experimentally examining whether different role models positively affect self-perception and reduce the effects of a negative stereotype, a small step in breaking the cycle of the negative perception of Europeans towards Roma.

## Supporting information

S1 Fig Experimental locations. (PDF)

S1 Table Randomization at the community/municipal level. (PDF)

S2 Table Selected Census 2011 municipality characteristics. (PDF)

S3 Table Demographic characteristics of subjects. (PDF)

S1 Appendix Background questionnaire. (PDF)

S2 Appendix Experimental task. (PDF)

S1 Dataset (XLSX)
