# Supplementary material for: Behavioural challenges of minorities: Social identity and role models
Source: PLoS One. 2019 Jul 26;14(7):e0220010. doi: 10.1371/journal.pone.0220010 (PMC6660091; doi:10.1371/journal.pone.0220010)
Supplement: S1 Fig — (PDF) [file pone.0220010.s001.pdf]

S1 Fig Experimental locations

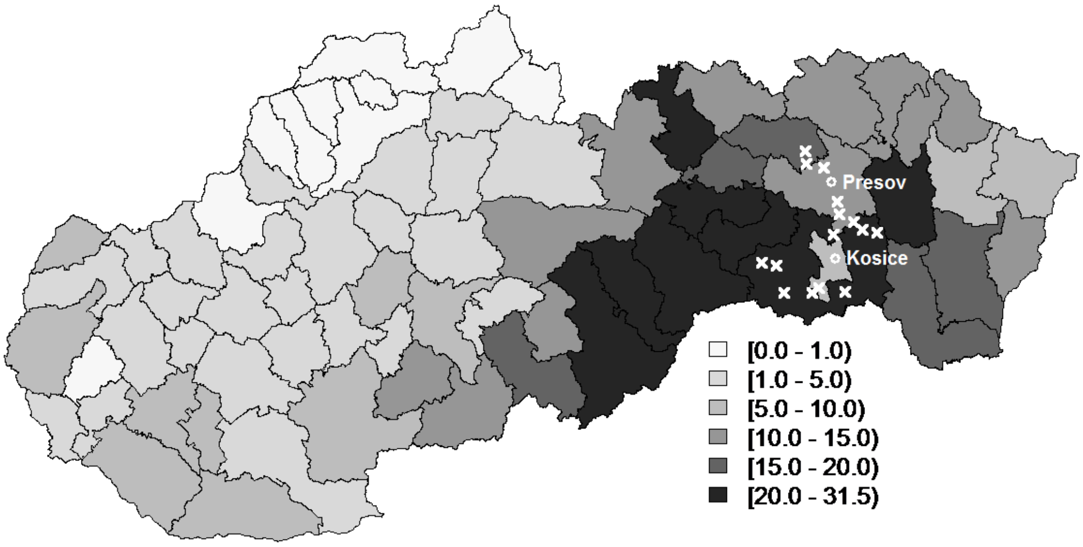

Note: The thematic map shows the distribution of the proportion of people with attributed Roma ethnicity (in %). The 'x' symbols indicate geographic locations of the experimental municipalities. Source of data: Mušinka A, Škobla D, Hurrle J, Matlovičová K, Kling J. Atlas of Roma Communities in Slovakia 2013. Bratislava, Slovakia: UNDP Europe and the CIS, Bratislava Regional Centre; 2014.
