# Supplementary material for: Behavioural challenges of minorities: Social identity and role models
Source: PLoS One. 2019 Jul 26;14(7):e0220010. doi: 10.1371/journal.pone.0220010 (PMC6660091; doi:10.1371/journal.pone.0220010)
Supplement: S1 Table — (PDF) [file pone.0220010.s002.pdf]

S1 Table Randomization at the community/municipal level

Characteristic	Sample (1)	Eastern Slovakia (2)	Difference (3)	Norm. Diff (4)
Access to water main (%)	52.0	52.5	−0.5	−0.009
	(7.2)	(1.9)	(7.4)	
Access to other source of water (%)	25.0	21.3	3.7	0.076
	(6.2)	(1.6)	(6.4)	
Without access to water (%)	13.9	10.9	3.8*	0.083
	(4.5)	(1.2)	(4.7)	
Public sewerage system usage (%)	30.5	20.5	10.0	0.191
	(7.0)	(1.6)	(7.1)	
Drain wells usage (%)	24.0	24.3	−0.3	−0.007
	(4.9)	(1.6)	(5.1)	
No access to sewerage system (%)	44.9	54.3	−9.4	−0.173
	(6.3)	(1.9)	(6.6)	
Electricity Usage (%)	91.7	92.5	−0.8	−0.029
	(3.6)	(0.9)	(3.7)	
Gas Usage (%)	20.6	13.6	7.0	0.162
	(6.1)	(1.3)	(6.2)	
Public street lights availability (%)	91.4	89.7	1.7	0.050
	(3.7)	(1.2)	(3.9)	
Heating by gas (%)	5.9	5.8	0.1	0.005
	(2.2)	(0.7)	(2.3)	
Heating by wood coal (%)	97.3	93.1	4.2	0.217
	(1.4)	(0.8)	(1.6)	
Kindergardens in the municipality (No.)	1.0	0.9	0.1*	0.333
	(0.0)	(0.0)	(0.0)	
Dist. to 1-4 grades primary school (km)	4.0	3.3	0.7	0.170
	(0.3)	(0.2)	(0.3)	
Dist. to 1-9 grades primary school (km)	5.0	5.1	−0.1	−0.025
	(0.3)	(0.2)	(0.3)	
Special primary schools in the municipality (No.)	0.3	0.2	0.1	0.156
	(0.1)	(0.0)	(0.1)	
Dist. to special primary school (km)	7.9	9.3	−1.4	−0.125
	(1.5)	(0.3)	(1.5)	
Dist. to the train stop (km)	9.2	10.1	−0.9	−0.054
	(2.3)	(0.5)	(2.4)	
Dist. to the general practitioner (km)	0.8	3.9	−3.1***	−0.665
	(0.3)	(0.2)	(0.4)	
Dist. to the pediatrician (km)	1.2	5.2	−4.0***	−0.662
	(0.5)	(0.2)	(0.5)	
Dist. to th gynecologist (km)	11.4	9.9	1.5	0.098
	(2.4)	(0.3)	(2.4)	

Notes: This table shows the *ex ante* balance in the characteristics of municipalities chosen for experiments. The upper part of the table reports characteristics at municipal level, the lower part at the municipal level. Approximative (permutation) Wilcoxon-Mann-Whitney Test significance level indications: $^{***}p < 0.01$,$^{**}p < 0.05$,$^{*}p < 0.10$.
