# Supplementary material for: Behavioural challenges of minorities: Social identity and role models
Source: PLoS One. 2019 Jul 26;14(7):e0220010. doi: 10.1371/journal.pone.0220010 (PMC6660091; doi:10.1371/journal.pone.0220010)
Supplement: S2 Table — (PDF) [file pone.0220010.s003.pdf]

S2 Table Selected Census 2011 municipality characteristics

Characteristics	Sample Municipalities (1)	Eastern Slovakia (2)	Slovakia (3)
Average age (years)	33.5	37.1	38.9
Single (%)	50.0	44.4	42.3
Married (%)	36.3	40.2	41.0
Slovak ethnicity/nationality (%)	78.4	85.9	86.8
Roma ethnicity/nationality* (%)	12.4	5.5	2.1
Slovak mother tongue (%)	70.5	81.6	84.9
Roma mother tongue (%)	17.8	7.2	2.5
Roman Catholics (%)	76.8	64.4	69.4
People with no religion (%)	8.8	9.5	15.0
Primary education level (%)	20.6	16.9	15.4
Secondary education level (%)	43.5	51.3	54.2
Tertiary education level (%)	11.2	12.8	14.3
No education (%)	24.7	19.0	16.1
Emplyed / self-employed (%)	68.2	75.9	82.1
Unemployed (%)	30.6	23.0	16.8
Retired (%)	13.8	18.0	19.7
Children + students (%)	30.9	26.3	23.3

*Nationality in context of ethnicity. Self-reported Roma ethnicity based on Census 2011 data (2.1 % at national level) is significantly lower than share of people with attributed Roma ethnicity (Mušinka A, Škobla D, Hurrle J, Matlovičová K, Kling J. Atlas of Roma Communities in Slovakia 2013. Bratislava, Slovakia: UNDP Europe and the CIS, Bratislava Regional Centre; 2014).
