# Supplementary material for: Behavioural challenges of minorities: Social identity and role models
Source: PLoS One. 2019 Jul 26;14(7):e0220010. doi: 10.1371/journal.pone.0220010 (PMC6660091; doi:10.1371/journal.pone.0220010)
Supplement: S3 Table — (PDF) [file pone.0220010.s004.pdf]

S3 Table Demographic characteristics of subjects

Characteristics	All obs. (1)	C (2)	T1 (3)	T2 (4)	T3 (5)	C − T1 (6)	C − T2 (7)	C − T3 (8)	T1 − T2 (9)	T1 − T3 (10)	T2 − T3 (11)
Children's characteristics											
Age	13.02	13.28	12.75	13.32	12.76	0.53	−0.04	0.52	−0.57*	−0.01	0.56
	(0.12)	(0.25)	(0.21)	(0.26)	(0.24)	(0.33)	(0.36)	(0.35)	(0.33)	(0.32)	(0.35)
						[0.16]	[−0.01]	[0.15]	[−0.17]	[0.00]	[0.16]
Male [%]	49.36	41.30	45.28	60.00	51.00	−3.98	−18.70**	−9.70	−14.72**	−5.72	9.00
	(2.52)	(5.13)	(4.83)	(5.03)	(5.00)	(3.10)	(3.21)	(3.17)	(3.06)	(3.01)	(3.13)
						[−0.06]	[−0.27]	[−0.14]	[−0.21]	[−0.08]	[0.13]
Regular primary [%]	66.50	61.96	76.42	66.67	60.00	−14.46**	−4.71	1.96	9.75	16.42**	6.67
	(2.38)	(5.06)	(4.12)	(4.81)	(4.90)	(2.99)	(3.16)	(3.14)	(2.92)	(2.90)	(3.07)
						[−0.22]	[−0.07]	[0.03]	[0.15]	[0.25]	[0.10]
Household/parents' characteristics											
Household income [EUR/month]	409.18	407.73	389.83	397.27	442.11	17.90	10.45	−34.38	−7.44	−52.27	−44.83
	(13.14)	(28.37)	(20.65)	(26.85)	(29.05)	(35.09)	(39.06)	(40.60)	(33.87)	(35.64)	(39.56)
						[0.07]	[0.04]	[−0.11]	[−0.03]	[−0.19]	[−0.15]
Parents married [%]	70.31	73.21	67.80	67.86	72.41	5.42	5.36	0.80	−0.06	−4.62	−4.56
	(3.02)	(5.92)	(6.08)	(6.24)	(5.87)	(4.43)	(4.52)	(4.41)	(4.49)	(4.38)	(4.47)
						[0.08]	[0.08]	[0.01]	[0.00]	[−0.07]	[−0.07]
Father's education = primary [%]	62.95	60.00	65.52	66.04	60.34	−5.52	−6.04	−0.34	−0.52	5.17	5.69
	(3.23)	(6.61)	(6.24)	(6.51)	(6.42)	(4.62)	(4.76)	(4.65)	(4.64)	(4.52)	(4.67)
						[−0.08]	[−0.09]	[0.00]	[−0.01]	[0.08]	[0.08]
Employed (regular or community work) [%]	45.85	46.43	45.76	50.00	41.38	0.67	−3.57	5.05	−4.24	4.38	8.62
	(3.29)	(6.66)	(6.49)	(6.68)	(6.47)	(4.62)	(4.72)	(4.64)	(4.63)	(4.55)	(4.64)
						[0.01]	[−0.05]	[0.07]	[−0.06]	[0.06]	[0.12]

Notes: This table shows the *ex post* balance in the demographic characteristics of subjects across treatments. "C" refers to the control, "T1" the Roma salient treatment, "T2" Roma role model treatment and "T3" non-Roma role model treatment. Means (Age and Household income) and percentages (all other characteristics) reported in columns (1) - (5), differences in means/proportions including t-test/Fisher's exact test in columns (6) - (11). SE estimates in parentheses, normalized differences in square brackets. Normalized differences are calculated using the formula as in Imbens & Wooldridge (2009). A rule of thumb is that if normalized difference exceeds 0.25 in absolute value, linear regression methods tend to be sensitive to the specification. Significance level indications: $^{***}p < 0.01$, $^{**}p < 0.05$, $^{*}p < 0.10$.
