# Supplementary material for: Behavioural challenges of minorities: Social identity and role models
Source: PLoS One. 2019 Jul 26;14(7):e0220010. doi: 10.1371/journal.pone.0220010 (PMC6660091; doi:10.1371/journal.pone.0220010)
Supplement: S1 Appendix — (PDF) [file pone.0220010.s005.pdf]

(C) Identity not revealed (Control)

No.	QUESTION	RESPONSE
①	FAVOURITE **FOOD** ———→	
②	PREFER WALKING **UPSTARS** OR **DOWNSTARS**?	☐ UPSTAIRS<br>☐ DOWNSTAIRS
③	FAVOURITE **COLOUR** ———→	
④	DO YOU PREFER **WINTER** OR **SUMMER**?	☐ WINTER<br>☐ SUMMER
⑤	WHAT IS THE CAPITAL OF **FRANCE**?	☐ LONDON<br>☐ PARIS<br>☐ DON’T KNOW

(T1) Roma Salient

No.	QUESTION	RESPONSE
①	FAVOURITE **FOOD** ————→	
②	PREFER WALKING **UPSTARS** OR **DOWNSTARS**?	☐ UPSTAIRS<br>☐ DOWNSTAIRS
③	FAVOURITE **COLOUR** ———→	
④	DO YOU PREFER **WINTER** OR **SUMMER**?	☐ WINTER<br>☐ SUMMER
⑤	WHAT IS THE CAPITAL OF **FRANCE**?	☐ LONDON<br>☐ PARIS<br>☐ DON'T KNOW
⑥	YOUR **NATIONALITY**	☐ ROMA<br>☐ SLOVAK<br>☐ HUNGARIAN<br>☐ OTHER: ....
⑦	**LANGUAGE** SPOKEN MOST FREQUENTLY AT HOME ——→	
⑧	DO YOUR GRANDPARENTS SPEAK ANY OTHER **LANGUAGE** OTHER THAN ROMA?	☐ YES<br>☐ NO

(T2) Roma Role Model

No.	QUESTION	RESPONSE
①	A POET **VLADIMÍR OLÁH** ESTABLISHED WHICH ASSOCIATION?	☐ ROMA BEE MOTHER<br>☐ SLOVAK BEE MOTHER<br>☐ DON'T KNOW
②	AN ACTIVIST **DR JÁN CIBUĽA** WHO WAS NOMINATED FOR A NOBEL PEACE PRIZE AND WAS ALSO A PRESIDENT OF INTERNATIONAL ROMA UNION STUDIED AT HIGH SCHOOL IN:	☐ RIMAVSKÁ SOBOTA<br>☐ TRNAVA<br>☐ DON'T KNOW
③	**SILVIA ŠARKÖZIOVÁ** IS A MEMBERS OF GROUP	☐ GIPSY KINGS<br>☐ GYPSY DEVILS<br>☐ DON'T KNOW
④	**IGOR KMEŤO**, MEMBER OF KMEŤOBAND SANG A SONG:	☐ BUBA MARA<br>☐ Ó MAŇO<br>☐ DON'T KNOW
⑤	MAIDEN NAME OF THE SINGER **VĚRA BÍLÁ** WAS:	☐ VĚRA GIŇOVÁ<br>☐ VĚRA OLÁHOVÁ<br>☐ NEVIEM
⑥	HOW MANY MEMBERS DOES THE BAND **GIPSY KAJKOS** HAVE?	☐ FEWER THAN 4<br>☐ MORE THAN 4<br>☐ DON'T KNOW

(T3) Non-Roma Role Model

No.	QUESTION	RESPONSE
①	IS **JUSTIN BIEBER** MORE THAN 18 YEARS OLD?	☐ YES<br>☐ NO<br>☐ DON'T KNOW
②	WHAT SOCCER CLUB DOES **CRISTIANO RONALDO** PLAY FOR?	☐ REAL MADRID<br>☐ FC BARCELONA<br>☐ DON'T KNOW
③	WHAT SOCCER CLUB DOES **MAREK HAMŠÍK** PLAY FOR?	☐ MANCHESTER UNITED<br>☐ SSC NAPOLI<br>☐ DON'T KNOW
④	IS **SHAKIRA** A PART OF A GROUP OR IS SHE A SOLO SINGER?	☐ PART OF A GROUP<br>☐ SOLO SINGER<br>☐ DON'T KNOW
⑤	THE SINGER **HELENA VONDRÁČKOVÁ** PERFORMED A HIT	☐ VYZNANIE (Declaration)<br>☐ DLOUHÁ NOC (Long Night)<br>☐ DON'T KNOW
⑥	HAS THE SINGER **KAREL GOTT** BEEN AWARDED „THE GOLDEN NIGHTINGALE" PRIZE MORE THAN 20 TIMES?	☐ YES<br>☐ NO<br>☐ DON'T KNOW
