# Supplementary material for: Behavioural challenges of minorities: Social identity and role models
Source: PLoS One. 2019 Jul 26;14(7):e0220010. doi: 10.1371/journal.pone.0220010 (PMC6660091; doi:10.1371/journal.pone.0220010)
Supplement: S2 Appendix — (PDF) [file pone.0220010.s006.pdf]

NUMERIC PUZZLES

EXAMPLE #1:

COUNT ZEROES (0) IN NUMBER:

12303204004

ANSWER: ……………………

EXAMPLE #2:

COUNT ZEROES (0) IN NUMBER:

021200231003200690

ANSWER: ……………………

①	HOW MANY PUZZLES DO YOU THINK YOU CAN SOLVE PROPERLY IN **1 MINUTE** ——————————→	
②	HOW MANY PUZZLES DO YOU THINK YOU CAN SOLVE PROPERLY IN **3 MINUTES** —————————→	

IN EACH OF THE FOLLOWING NUMERIC PUZZLES, COUNT ZEROES (0) IN NUMBER AND WRITE DOWN YOUR ANSWER. SOLVE THE PUZZLES IN THE ORDERING AS LISTED BELOW.

EXAMPLE:

12303204004	ANSWER: 4

---

1. | 2106205400427 | ANSWER:............ |
2. | 3906787391206710000 | ANSWER:............ |
3. | 870022028350338000 | ANSWER:............ |
4. | 8205014447900910000 | ANSWER:............ |
5. | 5900081308109990000 | ANSWER:............ |
6. | 184076028379002 | ANSWER:............ |
7. | 8082107820 | ANSWER:............ |
8. | 2027041620946290000 | ANSWER:............ |
9. | 60808440688762500000 | ANSWER:............ |
10. | 418700903865126000 | ANSWER:............ |
11. | 152681508274905000 | ANSWER:............ |
12. | 1200094030704 | ANSWER:............ |
13. | 723000905843504 | ANSWER:............ |
14. | 307078407116015 | ANSWER:............ |

15. 6073086004243690000 ANSWER:............

16. 391020750730230000 ANSWER:............

17. 338910093909084000 ANSWER:............

18. 4893007210609 ANSWER:............

19. 300631709084309000000 ANSWER:............

20. 8108301693009020 ANSWER:............

21. 16902950009 ANSWER:............

22. 92088700011500000000 ANSWER:............

23. 928990995480908 ANSWER:............

24. 6731980199090420000 ANSWER:............

25. 10023070685015 ANSWER:............

26. 2408030588054000 ANSWER:............

27. 500053231384 ANSWER:............

28. 11913761608 ANSWER:............

29. 86100408339 ANSWER:............

30. 246096123806 ANSWER:............

31. 804938514747000 ANSWER:............

32. 8586702600893050000 ANSWER:............

33. 619078005032 ANSWER:............

34. 60643600540222800 ANSWER:............

35. 59306290042001700 ANSWER:............

36. 40563069500 ANSWER:............

37. 4020302012007 ANSWER:............

38. 6450268092509 ANSWER:............

39. 963200433054096 ANSWER:............

40. 68007879055 ANSWER:............

41. 4724860972010 ANSWER:............

42. 90067008269062100 ANSWER:............

43. 2509195600170790000 ANSWER:............

44. 9500020714300 ANSWER:............

45. 4044740704009640 ANSWER:............

46. 8065283909700500000 ANSWER:............

47. 60067607793222000 ANSWER:............

48. 52087790609591 ANSWER:............

49. 6756024008038520000 ANSWER:............

50. 960043030601058 ANSWER:............

51. 405209633800810 ANSWER:............

52. 563006016070640000 ANSWER:............

53. 6070076236908050000 ANSWER:............

54. 6309465007 ANSWER:............

55. 465270051690000000 ANSWER:............

56. 840805060018669000 ANSWER:............

57. 330383147000096000 ANSWER:............

58. 5296058240100000000 ANSWER:............

59. 9720710209 ANSWER:............

60. 1018090500047770000 ANSWER:............

61. 4005087050669010 ANSWER:............

62. 25103009027910 ANSWER:............

63. 402650964440 ANSWER:............

64. 778050703010 ANSWER:............

65. 8068400000406020000 ANSWER:............
